# Vy-PER: eliminating false positive detection of virus integration events in next generation sequencing data

**DOI:** 10.1038/srep11534

**Published:** 2015-07-13

**Authors:** Michael Forster, Silke Szymczak, David Ellinghaus, Georg Hemmrich, Malte Rühlemann, Lars Kraemer, Sören Mucha, Lars Wienbrandt, Martin Stanulla, UFO Sequencing Consortium within the I-BFM Study Group, Andre Franke

**Affiliations:** 1 Institute of Clinical Molecular Biology, Christian-Albrechts-University of Kiel, Schleswig-Holstein, D-24105 Kiel, Germany; 2 Department of Computer Science, Christian-Albrechts-University of Kiel, Schleswig-Holstein, D-24118 Kiel, Germany; 3 Department of Pediatric Haematology and Oncology, Hannover Medical School, Lower Saxony, D-30625 Hannover, Germany

## Abstract

Several pathogenic viruses such as hepatitis B and human immunodeficiency viruses may integrate into the host genome. These virus/host integrations are detectable using paired-end next generation sequencing. However, the low number of expected true virus integrations may be difficult to distinguish from the noise of many false positive candidates. Here, we propose a novel filtering approach that increases specificity without compromising sensitivity for virus/host chimera detection. Our detection pipeline termed Vy-PER (**V**irus integration detection bY Paired End Reads) outperforms existing similar tools in speed and accuracy. We analysed whole genome data from childhood acute lymphoblastic leukemia (ALL), which is characterised by genomic rearrangements and usually associated with radiation exposure. This analysis was motivated by the recently reported virus integrations at genomic rearrangement sites and association with chromosomal instability in liver cancer. However, as expected, our analysis of 20 tumour and matched germline genomes from ALL patients finds no significant evidence for integrations by known viruses. Nevertheless, our method eliminates 12,800 false positives per genome (80× coverage) and only our method detects singleton human-phiX174-chimeras caused by optical errors of the Illumina HiSeq platform. This high accuracy is useful for detecting low virus integration levels as well as non-integrated viruses.

Two primary drivers for developing virus integration detection methods are the fields of disease therapy and disease etiology. In gene therapy and immunotherapy studies, a major consideration is the non-integration^1^ or at least the safe integration of a vector’s payload into the host genome^2^,^3^,^4^. In disease etiology, prominent examples of integrations into the host genome are the retroviruses human T-lymphotropic virus (HTLV) in adult T-cell leukemia^5^ and human immunodeficiency virus (HIV) in acquired immune deficiency syndrome (AIDS). Recent studies reported that integration of HIV at specific genomic locations leads to clonal expansion of virus-infected cells – slowing viral decay under combination antiretroviral therapy (cART) – and to cancer initiation^6^,^7^. Other etiologically important viruses that may integrate using different methods are hepatitis B virus (HBV) in liver cancer and human papillomavirus (HPV) in cervical, anal, oropharynx and other cancers^8^,^9^,^10^. Epstein-Barr virus (EBV) is associated with Burkitt’s lymphoma^11^ and is routinely used to immortalise cell lines^12^,^13^, but has also been reported to integrate into the host genome at very low frequencies^14^,^15^,^16^. Regardless of whether viruses are integrated into the host genome or not, one causal mechanism for cancer development is binding of virus proteins to the tumour suppressor *p53*, thereby inhibiting apoptosis. This mechanism is exploited for example by HBV^17^, HPV^18^, herpes simplex type 1^19^, measles^20^, or simian virus type 40^21^. Another causal mechanism was hypothesised when HBV integration throughout the human genome was detected in liver cancer^22^ and associated with genomic instability, putatively caused by fusion transcripts. A third causal mechanism was more recently suggested when HBV integration sites were found to recurrently cluster near genomic rearrangement sites and were therefore associated with chromosomal instability (chromothripsis)^23^,^24^,^25^. This discovery led to systematic and large-scale virus integration analyses of The Cancer Genome Atlas data^11^,^26^, and of data in many ongoing cancer studies including the childhood acute lymphoblastic leukemia (ALL) deep sequencing pilot study initiated by the German Federal Office for Radiation Protection (http://goo.gl/q7SaUZ). Childhood ALL is mostly classifiable by fusion genes^27^,^28^ and is usually associated with exposure to radiation^29^,^30^, like other childhood cancers^31^. The exact causes of ALL remain unknown. To our knowledge, there have been no previous studies on virus integrations in childhood ALL.

Virus integrations can be detected using Illumina paired-end sequencing (Fig. 1), if one read maps to the human genome and its paired end to a virus genome^23^,^24^. We analysed our childhood leukemia data accordingly using own scripts, as the data were too large for publicly available pipelines even on our high performance Linux cluster (928 cores with the hardware limit of 125 GB RAM per job). In addition, the pipelines that we tested were quite slow and did not detect virus integrations in benchmarking runs of our leukemia data. Our initial scripts identified a surprisingly large number of virus candidates. On closer inspection we noticed that many virus candidates were simply long stretches of unspecific short tandem repeats (STRs) or homopolymers (Table 1). These unspecific sequence stretches turned out to be identical in many virus genomes as well as the human genome. We therefore developed filters to eliminate false positives efficiently without compromising the sensitivity to detect true chimeric human/virus sequences. One solution to eliminate nearly all false positives would be to filter low-complexity reads with DUST^32^, as implemented for example in BLAST^33^ searches, or with PRINSEQ^34^. To avoid removing potentially informative reads (see Supplementary Results online), we prefer to filter reads by only considering the length of its longest STR. A second solution to eliminate false positives would be to cluster the virus/human chimera candidates within a genomic window as implemented in the VirusSeq pipeline^26^, but this method by definition loses the sensitivity to detect low-frequency events represented by singleton or low-frequency chimeras. A third method would be to obtain supporting information on potential viruses from unmapped read-pairs, as implemented in the VirusSeq and VirusFinder pipelines^35^, which is limited to the co-occurrence of a virus, not its integration into the host genome. Our aim for high sensitivity was motivated by the knowledge that for example HIV or HTLV has a tropism for T-lymphocytes with CD4 receptors^36^,^37^, leaving most other cell types uninfected, and that viruses such as EBV or HTLV integrate at seemingly random sites and are difficult to detect unless a major clonal expansion takes place within the cell population^14^,^15^,^16^,^37^. With respect to the efficient use of computer resources, the computationally expensive alignment of all read-pairs to the human genome should ideally be performed just once, i.e. in the context of a classical BWA-based next generation sequencing (NGS) pipeline (Fig. 1). The resulting specific and efficient analysis pipeline would allow us to routinely scan all of our future genome, transcriptome, and targeted next generation sequencing data for integrations of known viruses before further wet lab tests or computationally more expensive analyses are performed on selected samples.

## Material and Methods

### Whole genome sequencing of leukemia samples

In 2012 the German Federal Office for Radiation Protection initiated a multi-center deep sequencing pilot study on childhood ALL (Supplementary consortium data online). We sequenced the whole genomes of paired tumour and control samples from ten pediatric patients according to the Illumina TruSeq paired-end sequencing protocol. The tumours had characteristic genomic rearrangements detected by PCR^38^. Samples were taken at diagnosis in different hospitals. The tumour cell content was between 60% and 95%. Each tumour sample was sequenced to a minimal coverage of 80× using an average of eight HiSeq 2000 lanes. As in nearly all current leukemia studies, the control samples were collected from the same patients when they were in remission, defined by a tumour cell content (MRD, minimal residual disease) less than 0.01%. Each control sample was sequenced to a minimal coverage of 40× using an average of four lanes.

The paired tumour and control samples were collected within the International BFM-Study Group (I-BFM-SG) from Austria, France, and Germany. The patients were enrolled by the AIEOP-BFM study group (Austria, Germany) and the FRALLE study group (France). Informed consent for the use of spare specimens for research was obtained from study individuals, parents or legal guardians. The research project reported here was approved by the Ethics Committee of the Medical Faculty, Christian-Albrechts-University, Kiel, Germany. All methods were carried out in accordance with the approved guidelines. The biological samples and sequence data are not consented for access outside of our consortium.

### Whole genome sequencing protocol

Illumina TruSeq 2 × 101 bp paired end sequencing was performed on Illumina HiSeq 2000 and HiSeq 2500 instruments (Illumina, San Diego, CA). In brief, we used 1 μg of DNA per sample, which is fragmented by sonication to lengths of approximately between 250 and 650 bps before end-repair and ligation of Illumina sequencing adapters and molecular barcodes. The resulting libraries are each attached to random positions on a glass carrier plate (flowcell), and sequenced: Put simply, in the “first run”, the positions of all clusters on the glass plate are optically identified, and the first end of each library is sequenced to a length of 101 bps. In the second run, the library is sequenced from the second end to a length of 101 bps. Importantly, the position of each library on the flowcell is optically identified by the instrument and in rare cases a library position may be identified differently in the second run, leading to a chimeric sequencing artefact consisting of two different libraries.

### Bioinformatic availability and implementation

All Vy-PER scripts (**V**irus integration detection b**Y** **P**aired **E**nd **R**eads) are freely available at http://www.ikmb.uni-kiel.de/vy-per. The downloads include example scripts for a Linux cluster, human genome hg19 reference, NCBI virus genome references and benchmark data. The Vy-PER scripts are implemented in Python 2.7 with pysam v0.6, R, the Bioconductor^39^ packages quantsmooth^40^ and RColorBrewer, and bash scripts. Further prerequisites are BLAT^41^, BWA^42^, Phobos 3.3.12 (Christoph Mayer, http://www.rub.de/spezzoo/cm/cm_phobos.htm), and SAMtools^43^. For final filtering we used a Smith-Waterman implementation on the FPGA-based hardware RIVYERA S6-LX150 from SciEngines (www.sciengines.com), but for general users, Vy-PER also provides the option of using BLAT on a Linux cluster.

**Figure 1:**
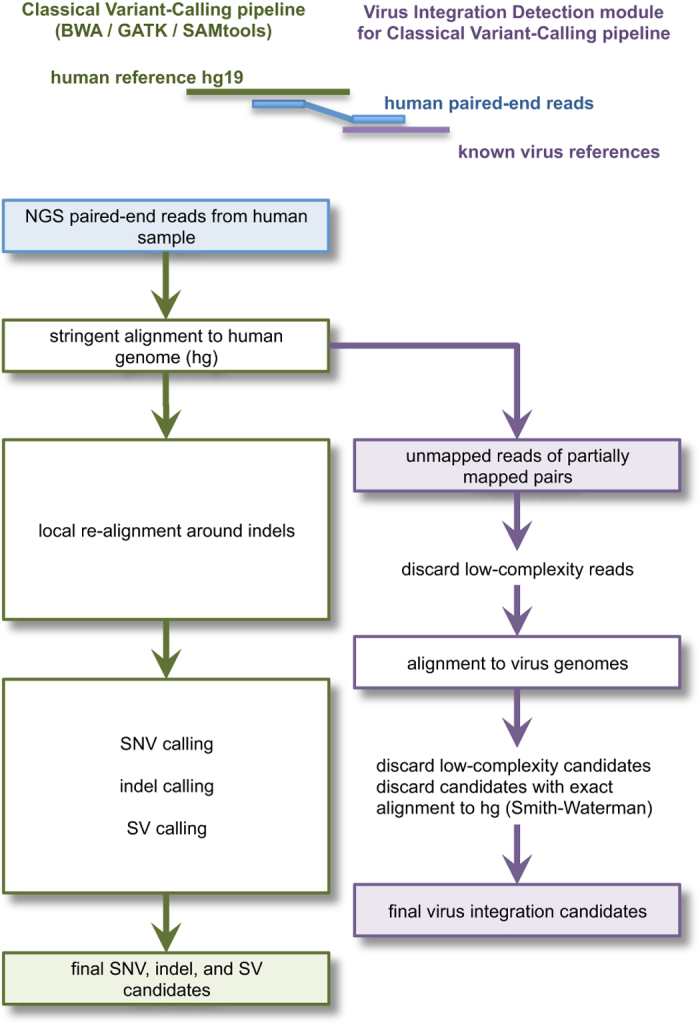
Detection of virus integrations into the human genome using paired-end sequencing. After the computationally expensive classical alignment of all paired-end reads to the human genome, the pipeline splits into classical variant calling and virus integration detection.

The bioinformatic workflow is summarised in Fig. 1 and described in detail in the Supplementary Methods online. In brief, the first step is the classical alignment of the human sequencing reads to the human genome prior to classical NGS variant calling, for example using BWA and SAMtools. This alignment is generally performed with stringent settings to keep run-time low and to map reads with high confidence for subsequent variant calling. However, a percentage of human reads will remain as “unmapped reads” that could not be aligned to the human reference. After this first step, the pipeline splits into two sub-pipelines that can be run in parallel if a computer cluster is available: (i) classical variant-calling, and (ii) virus integration detection. In the virus detection sub-pipeline, the first step is to extract paired-ends for which one read aligned to the human genome and the other end did not. The second step is to discard low-complexity reads with a long short tandem repeat or homopolymer. The third step is to test remaining reads for viral origin by alignment to known virus genomes. Some reads may map full-length or partially to a virus genome. In the fourth step, these candidate virus sequences are tested for low complexity, as a partial-length mapping result to a virus genome may consist only of a short tandem repeat or homopolymer (Table 1). In the final step, the remaining candidate sequences are tested for human origin using exact alignment to the human genome. This is computationally extremely expensive and can therefore usually be performed for only a few hundred sequences on a normal Linux computer cluster, hence the preceding multi-step filtering/testing process. Two sub-steps are used to speed up this exact alignment to the human genome: Each candidate is exactly aligned to its own small reference sequence window around the end of the read-pair that mapped to the human genome. Those candidates failing the first, local alignments are consecutively exactly aligned against the entire human genome. For exact alignment, BLAT is used with special settings (Supplementary Methods online), and for optional speed increase we use the Smith-Waterman implementation on FPGA hardware.

### Bioinformatic pipeline comparison

We here also compare the virus integration detection by Vy-PER with several recently published pipelines using their default settings or recommendations: the VirusSeq pipeline^26^, the VirusFinder pipeline v.2.0^35^, and the ViralFusionSeq pipeline revision1289^44^. As the VirusFinder pipeline does not include virus reference sequences on its project homepage, we followed the authors’ recommendations and downloaded the RINS reference sequences^45^. Out of interest, we also used VirusSeq’s gibVirus reference sequences (http://gib-v.genes.nig.ac.jp/) for VirusFinder. The benchmark timings were obtained by running each pipeline on a single 16-core linux computer node with 120 GB RAM, except for Vy-PER which only required a single core and less than 10 GB RAM; all file input/output was performed via a 10 Gbit/s network to a central EMC Isilon X200 storage cluster. We included the Amazon EC2 cloud-based instance of SURPI^46^ for detecting the presence of viruses without the ability to detect their integration. We tested SURPI v1.0.18 (July 10, 2014) using the EC2 Amazon Machine Image ami-a28f4cca on an i2.4xlarge instance in Amazon’s US-East region, as described in the SURPI protocol.

### Example data for the bioinformatic pipeline comparison

Download links to all four example data sets are available at http://www.ikmb.uni-kiel.de/vy-per.

*NA12878V* is a synthetic data set that we constructed by extracting 10,000 read-pairs from a 1000 Genomes Project^47^ Illumina paired-end whole genome sequencing BAM file for individual NA12878^48^ and adding 11,205 read-pairs from multiple individuals. The 11,205 read-pairs consist of 9,832 human/human pairs and 1,373 human/virus chimeras: The human/virus chimeras comprise 1,334 enterobacteria phage phiX174 chimeras, 2 enterobacteria phage M13 chimeras, and 37 human herpes virus type 3 (HHV-3) chimeras. The 9,832 human/human read-pairs include sequence stretches with similarities to virus sequence stretches, in order to test whether the benchmarked pipelines incorrectly detect herpes viruses (false positives). For patient confidentiality reasons, the original FASTQ sequence files and sequences in analysis or results files are removed from the publicly downloadable data. The paired-ends were aligned to hg19 using BWA and SAMtools. Then the Vy-PER pipeline was run as described.

*L526401A liver cancer sample (RNA)*. As our patient data cannot be publicly released, we here demonstrate the detection of known HBV integrations into liver cancer cell genomes employing publicly available Illumina paired-end (2 × 50 bp) transcriptome data^26^ with known HBV integrations. Before Vy-PER was run, the 53,060,622 paired-ends were aligned to hg19 using BWA and SAMtools, which required 3 h 2 minutes on a 16-core node. Then the Vy-PER pipeline was run as described.

*198T* and *268T* comprise publicly available “cleaned” Illumina paired-end (2 × 90 bp) whole genome tumour data with known HBV integrations, from two liver cancer patients in a study of 88 liver cancer patients^23^. The original raw sequences do not seem to be publicly available. We selected subsets of *198T* and *268T* sequencing data to test the sensitivity of our method and of other approaches at ultra low virus integration content. The subsets consist of 82,708,061 (198T) and 82,450,511 (268T) read-pairs, respectively. Before the Vy-PER run, alignment to hg19 was performed using BWA and SAMtools, which required 3 hours 22 minutes (198T), and 3 hours 45 minutes (268T), respectively. Then the Vy-PER pipeline was run as described.

## Results

### No evidence of somatic virus integration in childhood acute lymphoblastic leukemia samples with genomic rearrangements

**Figure 2:**
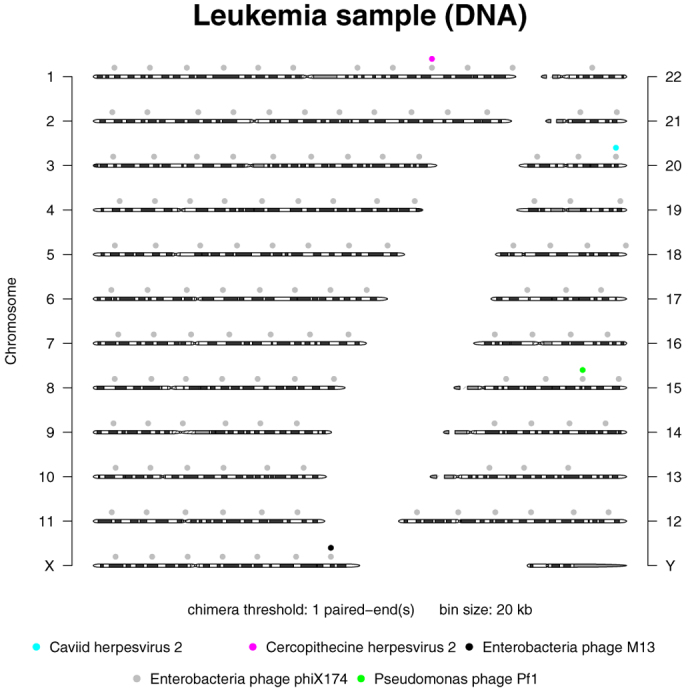
Vy-PER ideogram summary plot. Negative example without true virus integrations: Patient genome sequenced with 40× coverage and analysed with highest sensitivity, eliminating 6400 false positives and leaving three unsupported singletons (pseudomonas phage, caviid herpes, cercopithecine herpes) which we manually eliminated as alignment artefacts. PhiX and M13 originated from the 1% Illumina sequencing library control spike-in. The plot shows integration sites down to single chimeras.

Using our Vy-PER method, we searched for virus integrations in 10 tumour samples that were sequenced to a minimal coverage of 80×, and in 10 matched normal samples from the same patients that were sequenced to a minimal coverage of 40×. Before final filtering (see Supplementary Methods online), we detected on average 1600 virus candidates per lane (12,800 per 80× genome). On manual inspection, most candidates appeared to be false positives. Table 1 shows a representative snapshot of false positive virus candidates with intriguingly plausible viruses, e.g. the common herpes viruses. However, their candidate sequences are non-specific STR-rich or homopolymer-rich sequences. It can be argued that some herpes types are known to integrate into human telomeres^49^, but on the other hand, some herpes virus genomes simply have homologies to the human telomeres^50^. When we aligned a number of full-length HiSeq reads from herpes candidates to the expected human genome sequence window using an interactive DIALIGN-based tool^51^, we found that these reads were in fact from the human genome. Our alignment exercise suggested that a more accurate and more sensitive alignment of the virus candidates to the host genome would be an effective filter to eliminate false positives. Therefore, we implemented the final filtering of candidates (Fig. 1). The STR-based filtering of virus candidate sequences and exact alignment of remaining sequences to the expected human genome window cut the number of false positive virus candidates to only 12 candidates per 80× genome, on average. The final exact alignment to the whole human genome eliminated most of these remaining few sequences. In the end, no true virus/human chimeras were detected (Fig. 2, Table 2) even at the lowest reporting threshold of 1 paired-end in 20 M bps. The only reported candidates were sparsely distributed phiX174 chimeras, a few singletons, and a cluster of three enterobacteria phage M13 chimeras in the remission sample of patient 8 but not in the tumour. The phiX chimeras are probably optical positioning errors of the HiSeq platform when the sequencing of the second read of a paired-end run is performed. PhiX libraries are technical controls for base calling on the Illumina platforms. They are spiked into the completed sample libraries as a fraction of about 1% of the total libraries. The phiX libraries should not normally ligate with sample libraries, as both libraries are blunt-ended. The fraction of phiX chimeras in the ALL samples comprised about 0.0001% of the total paired-ends, or about 160 chimeras per lane. Enterobacteria phage M13 is used for bacterial cloning, as in the commercial production of phiX. The M13 singletons and the small M13 cluster that we detected are a trace contaminant of the Illumina phiX174 libraries. This suggests that our STR-filtering and host-genome re-alignment strategy is very promising for the elimination of human sequence reads and the preservation of virus/host chimeras, even down to singletons.

### Bioinformatic pipeline comparison

*Childhood acute lymphoblastic leukemia whole genome data from one HiSeq lane.* To compare run times between different pipelines (Table 3), we selected one HiSeq 2000 lane of data from one childhood ALL sample and ran each pipeline on an identical 16-core computer, except for Vy-PER which only requires a single core. One lane is approximately equivalent to a 10× covered genome. Analysis of the full genomic data was not feasible due to the computational duration and the unavailability of sufficient computing resources. After the classical BWA/SAMtools alignment to hg19, which required 13.5 hours, Vy-PER completed within 1 hour. VirusFinder required only 12.75 hours because it did not detect any viruses, but each detected virus would roughly add another 13 hours due to the renewed alignment of all reads to a combined hg19/virus reference sequence. ViralFusionSeq required 19 hours, the greater part of which is due to BWA-SW alignment which can be slower than BWA alignment. VirusSeq ran for 195 hours, mainly due to its use of the Mosaik aligner^52^, and failed in its final step. We could not test the Amazon EC2 cloud based instance of SURPI on this data set, due to severe performance issues. With respect to their detection results, VirusFinder, ViralFusionSeq and VirusSeq did not report human/virus chimeras in the leukemia data set (Table 4), ignoring the M13 and phiX seen by Vy-PER. VirusSeq incorrectly reported non-integrated carp herpesvirus sequences (Table 5). VirusFinder with the recommended RINS database incorrectly reported human herpesvirus type 5 (HHV-5) which we analysed and found to be the harmless sequencing control library component M13. With the alternative gibVirus database, VirusFinder reported the non-integrated virus J02482M10348M10379M10714M10749M10750M10866 (Table 5) and its *de-novo* assembled contig which our subsequent manual BLAST search identified as phiX174.

*NA12878V synthetic genome data.* The known human/virus chimeras were reported by Vy-PER but no other pipelines: Vy-PER reported 24 × human herpesvirus 3 (HHV-3), 1185 × phiX, and 2 × M13 chimeras where the human reads were aligned with a mapping quality of at least 20. HHV-3 and phiX were reported as non-integrated viruses by SURPI and VirusFinder, but not by ViralFusionSeq or VirusSeq (Table 5). The known M13 phage was detected by no other pipeline. The herpes-like decoy sequences in this example data did not trigger any false positive calls by any pipeline except for Vy-PER, which reported a single caviid herpesvirus chimera. SURPI and VirusFinder (gibVirus database) reported some false positive non-integrated enterobacteria phages. It should be noted that SURPI removes low-complexity reads as the first step, possibly over-aggressively, as true reads are removed which can be aligned by BWA (Supplementary Results online).

**Figure 3:**
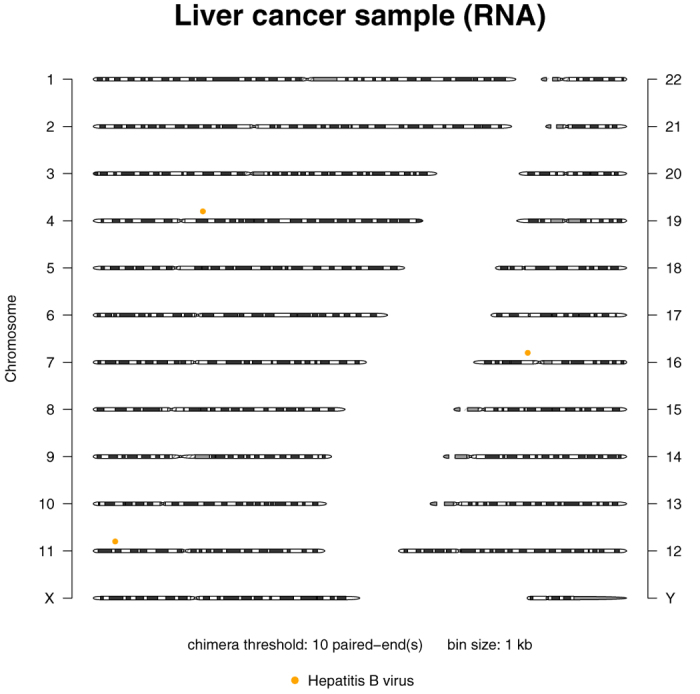
Vy-PER ideogram summary plot. Positive example with true virus integrations: Publicly available whole transcriptome liver cancer data analysed with default sensitivity, showing HBV candidate loci on chromosomes 4, 11 and 16. The plot only shows integrations supported by 10 or more chimeras.

**Figure 4:**
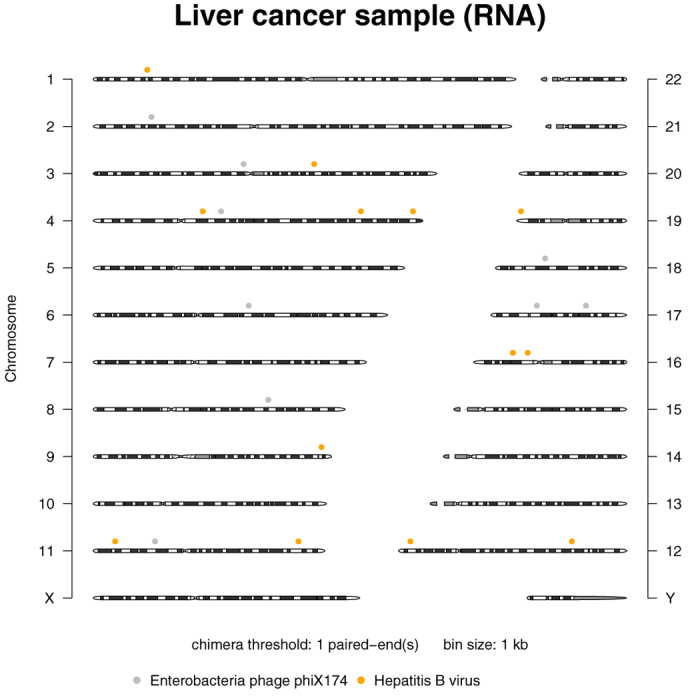
Vy-PER ideogram summary plot. HBV integration loci into the liver cancer genome detected at low stringency (threshold: 1 chimera), also showing detected phiX singletons.

*L526401A liver cancer sample (RNA).* The known HBV integration loci were reported by Chen and colleagues^26^ and we reproduced these loci using their VirusSeq pipeline (Supplementary Table S1 online). All reported HBV loci were also detected by Vy-PER. Figure 3 displays the virus candidate loci in the genome that were detected by Vy-PER with the stringent default setting of at least ten supporting chimeras, i.e. at least ten virus/host paired-ends are required to support a virus integration locus, and Table 6 lists the virus candidate loci in 1000 bp bins. Note that there are three integration loci on chromosome 16, one more than reported by VirusSeq. However, there is no split read to support this last locus, only the respective paired read that was aligned to hg19. In transcriptome data, a paired-end library may conceivably span two or more exons, which could lead to an additional candidate locus that is 4000 bp distant from the true integration locus. Table 7 shows the number of virus candidates reported by Vy-PER if singletons are enabled, and Fig. 4 shows the corresponding candidate loci. The VirusSeq pipeline includes gene annotation of the integration loci. However, the annotation is occasionally misleading, here for the integration locus chr4:63647816-63648816 which is 1.5 megabases distant from the 3’ end of the *TECRL* gene. This locus is nevertheless annotated as “*TECRL*/3-prime”, which the VirusSeq authors have also copied into their publication^26^. The actual locus that VirusSeq computed is correct, but it is located in a gene desert. The nearest gene, *LPHN3*, is less than half the distance to *TECRL*. The ViralFusionSeq pipeline reported 10 clipped fusion sequences of which most are duplicates, but did not report their integration loci (Supplementary Table S2 online). Confusingly, the 100 bp fusion sequences reported by ViralFusionSeq are a tandem repeat of identical 50 bp reads. A BLAT search for the integration locus did not place any of these fusion reads correctly, due to their short length of only 50 nucleotides and the unavailable paired-end information. Only one such read aligned to the correct locus on chromosome 11, but the alternative locus on chromosome 20 was ranked higher in the BLAT search. The VirusFinder pipeline did not report any virus integrations at all (Table 4 and Supplementary Table S1 online). Finally, no other pipeline reported the phiX chimeras seen by Vy-PER, but VirusFinder reported non-integrated phiX sequences.

*198T liver cancer genome data.* The liver cancer tissue sample harbours seven known human/HBV breakpoints^23^. We tested the sensitivity of all pipelines using a low-coverage subset of the whole genome data (Table 3). The only pipeline that was able to detect HBV in this data subset was Vy-PER. The virus integration event is detected by merely a single human/HBV chimera, but its locus is nevertheless validated by the known data^23^. There are no phiX chimeras in this data set, because the publicly deposited data has been “cleaned”. Looking at false positives, VirusSeq again incorrectly reported a non-integrated carp herpesvirus (Table 5).

*268T liver cancer genome data.* The liver cancer tissue sample harbours four known human/HBV breakpoints^23^. Only Vy-PER and ViralFusionSeq detected HBV integration in the low-coverage data subset of the full whole genome data. Vy-PER reported a single human/HBV chimera with a confidently mapped human read and a split human/virus read, which is validated by the known data^23^. ViralFusionSeq reported seven human/HBV fusion reads but did not resolve the coordinates (Supplementary Table S2 online). Manual BLAT and BLAST searches mapped four fusion reads to the same locus that was detected by Vy-PER, and the remaining three fusion reads to two further known loci (Supplementary Results online). We manually identified one HBV insertion to have microhomologies of four shared nucleotides between virus and host at the first end of the insertion (detected by Vy-PER), and of five shared nucleotides at the other end (detected by our manual searches for the fusion reads reported by ViralFusionSeq) (Supplementary Results online). The third insertion locus even showed a microhomology of 13 nucleotides. With respect to false positives, VirusSeq again incorrectly reported a non-integrated carp herpesvirus (Table 5).

## Discussion

Virus integrations into the host genome of cancer tissue can be detected using paired-end whole genome or transcriptome sequencing data. However, the low number of expected true virus integrations may be difficult to distinguish from the noise of many false positive candidates if aiming to increase sensitivity. For example, some viruses target only a fraction of cell types, such as the human herpesvirus-6 (HHV-6) which has a tropism for CD4+ T-lymphocytes^53^. We therefore analysed false positive virus integration candidates and developed accurate filtering methods and efficient Python and R scripts that can be integrated into any BWA-based next-generation sequencing analysis pipeline. The key strategies to eliminate false positives are to (a) remove reads consisting mainly of unspecific STRs or homopolymers, (b) remove virus candidates with sequences consisting mainly of unspecific STRs or homopolymers, (c) make sure that virus candidates do not align to the host genome, using highly accurate and sensitive alignment tools such as BLAT with its most sensitive setting or optionally Smith-Waterman, and (d) test for supporting candidates, as singletons may be artefacts. Finally, phiX and M13 are Illumina’s sequencing platform control spike-ins and not true virus infections. The optional FPGA-based Smith-Waterman alignments to the complete human genome are useful if FPGA hardware is available. Even without this optional stage, only quite a small number of false positives remain after using BLAT to align virus candidates to a reduced human genome sequence window of e.g. length 500 bps. In a clinical setting, it is not a practical option to use BLAT with its most sensitive settings to align candidates to the entire human genome, as this may take hours or days, but for pure research the run time is less important. In this context it is worth noting that BLAST uses DUST-based masking and does not align some human reads (Supplementary Results online), making BLAT or Smith-Waterman more suitable for this specific task.

The results shown here demonstrate that Vy-PER is sensitive, specific and efficient enough to be used as a first pipeline for routinely scanning all NGS data sets for virus integrations. The efficiency of Vy-PER is explained by its filtering design for speedy processing of genomic data that can be furthermore parallelised for multi-lane genomes (map-reduce approach), and – when integrated into a BWA based pipeline – by avoiding the duplication of any expensive whole genome alignment computations. Once viruses of concern have been found in a sample, a more detailed analysis with ViralFusionSeq, VirusFinder and VirusSeq can then be performed. These latter pipelines are still in their early days and fairly complicated to install and understand, but each has its own merits. All of them also perform a search for viruses that did not integrate into the host genome. VirusSeq annotates the integration loci with gene information and computes breakpoints. VirusFinder is supposed to do the same, but our tested version (version 2) did not compute any integrations. ViralFusionSeq reports only the human/virus fusion reads but not the integration locus. Whereas Vy-PER was designed with concise clinical reporting in mind, the other pipelines are more detailed and do not provide a summary graphic or table. Vy-PER uses the NCBI virus genomes which are sufficient for uniquely pinpointing a virus at species level, and the summary tables show whether some or all virus reads were unambiguously assigned to a single virus species (Table 2). Similarly, for example, TIPP (Taxon Identification and Phylogenetic Profiling, http://www.cs.utexas.edu/users/phylo/research/projects.html) dumbs down its level of detail as necessary, from strain to species or even to higher levels in the phylogeny, when ambiguous reads are encountered. However, the tested other pipelines currently still report viral strain information, which can give users a false sense of security, especially as there are vast homologies between the strains of a species, and we are unsure how correct these virus databases actually are. For example, the less experienced user may well be excited by the carp herpesvirus incorrectly reported in three out of five example data sets by one pipeline, or the human herpesvirus type 5 (instead of M13) incorrectly reported by another pipeline. No doubt each pipeline was developed for a specific type of virus and cancer data set, so it is probably worthwhile for a researcher to run a suspicious data set, i.e. one in which Vy-PER detected a virus, through each of these pipelines and to keep the potential problems in mind. It is for example remarkable that ViralFusionSeq detected viruses in only two of the five example data sets, but nevertheless detected three of the four fusion sequences in the low-coverage data subset of tumour sample 268T. Vy-PER only detected one of these fusions to be a virus integration event into the human host, because it searches only for chimeric paired-ends and not also for fusion sequences. Nevertheless, the scan for chimeras by Vy-PER was more sensitive than the approaches in other pipelines, correctly detecting the known chimeras in all five examples.

Surprisingly, Vy-PER is also able to detect non-integrated viruses. Taking the example of phiX (1% Illumina sequencing control library spike-in) and M13 (trace contamination of phiX library), the known presence of these non-integrated viruses is detected as chimeric virus/human read-pair artefacts. In our whole genome leukemia sequencing study, on average 160 phiX/human chimeras were detected per HiSeq lane (0.0001% of read-pairs). The chimeric sequencing artefacts can be interpreted as randomly sampled non-integrated virus sequences. Indeed, on the related topic of bacterial identification from shotgun sequencing, it was recently suggested^54^ that only 100 randomly sampled 100 bp Illumina reads from pure bacterial DNA could be sufficient to identify the bacterial strain with over 90% specificity. For detecting non-integrated pathogens, the sensitivity is better if all paired-end reads that could not be aligned to the human genome are scanned. The scan can be followed by our approach to eliminate false positives, which is not limited to chimeras or to viruses.

As a matter of speculation, future studies may find that the concept of a human-virus breakpoint at a single base pair is not appropriate for all viruses. On the one hand it is known that retroviruses integrate at a distinct breakpoint when an integrase binds to its preferred nucleotide motifs^55^ which are then duplicated and flank the integrated viral sequence^56^. However, other viruses, specifically HBV, may integrate using the non homologous end joining (NHEJ) pathway^57^,^58^ which implies a shared microhomology between virus and host at the integration site, as observed in our manual inspections of the integration sites in data set 268T. The NHEJ mechanism suggests that the HBV integration into the human genome may not be the driver event for genomic instability, but that the HBV integration may have occurred as a passenger event after double-stranded breakage of the human chromosome.

In the treatment of HIV infections, cART may be unable to completely eliminate HIV if replication competent proviruses are integrated into long lived or proliferating CD4+ cells. Of particular interest are the integration loci which are preferentially located in genes that promote cell proliferation^6^,^7^. Using Vy-PER, the integration loci in clonally expanded cell populations would be detectable either using normal paired-end shotgun sequencing or even better, using targeted sequencing like linker-mediated polymerase chain reaction^6^ or integration site loop amplification^7^. To speed up the bioinformatic analysis, the full virus genome reference could be replaced by just the HIV genome references.

Finally, the wealth of incidental metagenomic findings in human NGS data is highly exciting for potential pathogen detection, even when only microscopically low read numbers are available that our approach can detect and assign to a virus genome.

## Additional Information

**How to cite this article**: Forster, M. *et al*. Vy-PER: eliminating false positive detection of virus integration events in next generation sequencing data. *Sci. Rep.* **5**, 11534; doi: 10.1038/srep11534 (2015).

## Supplementary Material

Supplementary Information

Supplementary Tables

## Figures and Tables

**Table 1:** Typical false positive virus candidates before final filtering.

**Virus candidate sequence**	**Probable virus candidate**	**More likely explanation**
TTAGGGTTAGGGCTAGGGCTAGGGCTAGGGCTAGGGCTAGGGCTAGGGCTAGGGCT	Cyprinid herpesvirus 3	STR (human telomere)
CTCTCTCTCTCTCTCTCACACACACACACACACACACACACACACACACACACAC	Ictalurid herpesvirus 1	STRs
TTTTTTTTTTTTTTTTTTTTTTTTTTTTTTTTTTTTTTTTTTTTTTTTTA	Caviid herpesvirus 2	mainly homopolymer
TATATATATATATATATATATATATATTTTTTTTTTTTTTTTTTTTTTTT	Cotesia congregata bracovirus	STR and homopolymer
AACCCTAACCCTAACCCTAACCCTAACCCTAACCCTANCCCTAACCCTA	Human herpesvirus 6A	STR (human telomere)
TAACCCTAACCCTAACCCTAACCCTAGCCCTAACCCTAACCCTAACCCTA	Human herpesvirus 7	STR (human telomere)

**Table 2:** Top viruses in a leukemia patient sample after final filtering, low stringency (threshold: 1 chimera).

**Weighted candidates**	**Unambiguously aligned reads**	**Virus**	**ID**
1655.3	1655	Enterobacteria phage phiX174	NC_001422.1
1.0	1	Pseudomonas phage Pf1	NC_001331.1
1.0	1	Caviid herpesvirus 2	NC_011587.1
1.0	1	Enterobacteria phage M13	NC_003287.2
1.0	1	Cercopithecine herpesvirus 2	NC_006560.1

Weighted candidates: For reads that align ambiguously to two or more viruses, we consider the top 3 viruses, assigning the highest weight to the first virus and the lowest weight (e.g. 0.3) to the last virus; the weight for an unambiguous alignment is 1.0.

**Table 3:** Wall clock run time comparison between different bioinformatic pipelines for five examples.

	**Leukemia**	**NA12878V**	**L526401A**	**198T**	**268T**
Data type	WGS	WGS	RNA-Seq	WGS	WGS
HiSeq lanes	1.0	≈ 0.0001	≈ 0.33	≈ 0.5	≈ 0.5
Read length	2 × 101 bp	2 × mixed	2 × 50 bp	2 × 90 bp	2 × 90 bp
Read pairs	2.1 × 10^8^	2 × 10^4^	5.3 × 10^7^	8.2 × 10^7^	8.2 × 10^7^
SURPI on EC2					
fast	–	14.8 h	–	–	–
comprehensive	–	24.5 h	–	–	–
EC2 cost	–	$ 250.00	–	–	–
ViralFusionSeq	19.1 h	3 mins	4 h	12.3 h	12.4 h
VirusFinder					
RINS virus db	14.5 h	2.7 h	9.8 h	21.5 h	15.4 h
gibVirus db	12.8 h	2.7 h	13.7 h	20.1 h	12.4 h
VirusSeq	195 h	17 mins	14.7 h	57.6 h	58.3 h
**Vy-PER**	**1 h**	**7 mins**	**21 mins**	**19 mins**	**19 mins**

WGS whole genome sequencing, RNA-Seq whole transcriptome sequencing, EC2 Amazon elastic cloud computing. Wall clock times obtained on a 16 core computer, except for Vy-PER which only needed a single core of a 16 core computer and < 1 minute on the connected FPGA computer. The average number of WGS read pairs per HiSeq lane in our leukemia project was 1.65 × 10^8^.

**Table 4:** human/virus chimera detection comparison between different bioinformatic pipelines for five examples.

**Example (viruses)**		**ViralFusionSeq**	**VirusFinder (RINS)**	**VirusFinder (gibVirus)**	**VirusSeq**	**Vy-PER**
Leukemia B2265L8 (phiX)	TP	–	–	–	–	phiX
	FP	–	–	–	–	–
						
NA12878V (HHV3, M13, phiX)	TP	–	–	–	–	HHV3, M13, phiX
	FP	–	–	–	–	CHV2 (singleton)
						
L52640A (HBV, phiX)	TP	HBV	–	–	HBV	HBV, phiX
	FP	–	–	–	–	–
						
198T (HBV)	TP	–	–	–	–	HBV
	FP	–	–	–	–	–
						
268T (HBV)	TP	HBV	–	–	–	HBV
	FP	–	–	–	–	–

RINS VirusFinder-recommended database, gibVirus alternative database, TP true positive, FP false positive. phiX enterobacteria phage phiX174, HHV3 human herpesvirus 3, M13 enterobacteria phage M13, CHV2 caviid herpesvirus 2, HBV hepatitis B virus.

**Table 5:** Virus detection comparison between different bioinformatic pipelines for five examples, regardless of whether virus integration into the host genome was detected.

**Example (viruses)**		**SURPI on EC2**	**Viral Fusion Seq**	**VirusFinder (RINS)**	**VirusFinder (gibVirus)**	**VirusSeq**	**Vy-PER**
Leukemia (phiX, M13)	TP	not run	–	phiX	J0 (phiX)	–	phiX
	FP	not run	–	HHV5	DE3, P7	carp herpesvirus	–
							
NA12878V (HHV3, M13, phiX)	TP	HHV3, phiX	–	HHV3, phiX	J0 (phiX), HHV3	–	HHV3, M13, phiX
	FP	α3, f1, G4, phiK	–	–	S13	–	CHV2 (singleton)
							
L52640A (HBV, phiX)	TP	not run	HBV	HBV, phiX	HBV, phiX	HBV	HBV, phiX
	FP	not run	–	–	–	–	–
							
198T (HBV)	TP	not run	–	HBV	HBV	–	HBV
	FP	not run	–	–	–	carp herpesvirus	–
							
268T (HBV)	TP	not run	HBV	HBV	HBV	–	HBV
	FP	not run	–	–	–	carp herpesvirus	–

EC2 Amazon elastic cloud computing, RINS VirusFinder-recommended database, gibVirus alternative database, TP true positive, FP false positive. phiX enterobacteria phage phiX174, M13 enterobacteria phage M13, J0 J02482M10348M10379M10714M10749M10750M10866, HHV5 human herpesvirus 5, DE3 enterobacteria phage DE3, P7 enterobacteria phage P7, HHV3 human herpesvirus 3, α3 enterobacteria phage alpha3, f1 enterobacteria phage f1, G4 enterobacteria phage G4, phiK enterobacteria phage phiK, S13 bacteriophage S13, CHV2 caviid herpesvirus 2, HBV hepatitis B virus.

**Table 6:** Virus candidate loci in liver cancer sample after final filtering, high stringency (threshold: 10 supporting paired-ends)

**Chr**	**Start**	**End**	**Candidates**	**Virus**
4	63647816	63648816	16.0	Hepatitis B virus
4	63651319	63652319	10.0	Hepatitis B Virus
11	12711328	12712328	71.0	Hepatitis B Virus
16	31413359	31414359	44.0	Hepatitis B Virus
16	31414755	31415755	18.0	Hepatitis B Virus
16	31418770	31419770	10.0	Hepatitis B Virus

**Table 7:** Top viruses in liver cancer (RNA-Seq) after final filtering, low stringency (threshold: 1 chimera).

**Weighted candidates**	**Unambiguously aligned reads**	**Virus**	**ID**
181.0	181.0	Hepatitis B virus	NC_003977.1
7.0	7.0	Enterobacteria phage phiX174	NC_001422.1
