# Supplementary material for: Vy-PER: eliminating false positive detection of virus integration events in next generation sequencing data
Source: Sci Rep. 2015 Jul 13;5:11534. doi: 10.1038/srep11534 (PMC4499804; doi:10.1038/srep11534)

Supplementary Information:

Vy-PER: eliminating false positive detection of virus integration events in next generation sequencing data

Michael Forster[1,*], Silke Szymczak[1,2], David Ellinghaus[1], Georg Hemmrich[1], Malte Rühlemann[1], Lars Kraemer[1], Sören Mucha[1], Lars Wienbrandt[3], Martin Stanulla[4], the UFO Sequencing Consortium within the I-BFM Study Group[5], Andre Franke[1]

[1] Institute of Clinical Molecular Biology, Christian-Albrechts-University of Kiel, Schleswig-Holstein, D-24105 Kiel, Germany

[2] Current address: Institute of Medical Informatics and Statistics, University Medical Center Schleswig-Holstein, Campus Kiel, Schleswig-Holstein, D-24105 Kiel, Germany

[3] Department of Computer Science, Christian-Albrechts-University of Kiel, Schleswig-Holstein, D-24118 Kiel, Germany

[4] Department of Pediatric Haematology and Oncology, Hannover Medical School, Lower Saxony, D-30625 Hannover, Germany

[5] See the Author Contributions for details

* Corresponding author. Tel: +49 431 597-1028; Fax: +49 431 597-5788; m.forster@ikmb.uni-kiel.de

The first two authors contributed equally.

SUPPLEMENTARY CONSORTIUM DATA **2**
SUPPLEMENTARY METHODS: DETAILED BIOINFORMATIC WORKFLOW **3**
1. INITIAL STRINGENT ALIGNMENT TO HG19. 3
2. EXTRACTION OF PARTIALLY UNMAPPED READ-PAIRS 3
3. FILTERING OF READS WITH LESS THAN 30 BP NON-STR REGION. 3
4. ALIGNMENT TO ALL NCBI VIRUS GENOMES. 3
5. FINAL FILTERING OF FALSE POSITIVE VIRUS CANDIDATES. 4
6. OPTIONAL SMITH-WATERMAN ALIGNMENT TO HOST GENOME. 4
7. RESULTS FILES. 4
SUPPLEMENTARY RESULTS **5**
TRUE HUMAN SEQUENCE REMOVED BY DUST FILTERING IN SURPI AND BLAST 5
MICROHOMOLOGIES: THREE IDENTICAL STRETCHES BETWEEN HUMAN AND VIRUS REFERENCES IN THE 268T PATIENT SEQUENCE AT HBV INTEGRATION LOCUS CHR19:30297359 6
MANUAL CHECK OF VIRALFUSIONSEQ HBV FUSION READS FOR DATA SET 268T: 7
Fusion read 1 .... *7*
Fusion read 2 .... *8*
Fusion reads 3 and 7 (identical) *9*
Fusion read 4 .... *10*
Fusion reads 5 and 6 (identical) *10*

Supplementary consortium data

The paired tumour and remission genomes of the acute lymphoblastic leukemia patients were sequenced for the UFO sequencing consortium within the I-BFM Study Group. The sequencing consortium is currently coordinated by Prof Dr Andre Franke and Prof Dr Martin Stanulla.

The consortium comprises the following members (in alphabetical order):

- Department of Oncology, University Children's Hospital Zurich, Zurich, Switzerland.
- Department of Pediatrics, University Hospital Schleswig-Holstein, Kiel, Germany.
- Department of Pediatric Oncology, Hematology and Clinical Immunology, Heinrich Heine University, Düsseldorf, Germany.
- European Molecular Biology Laboratory, Genome Biology, Heidelberg, Germany.
- Federal Office for Radiation Protection, Oberschleißheim, Germany.
- Group Algorithmic Bioinformatics, Heinrich-Heine University, Düsseldorf, Germany.
- Institute of Clinical Molecular Biology, Christian-Albrechts-University of Kiel, Germany.
- Max Planck Institute for Molecular Genetics, Berlin, Germany
- Pediatric Hematology and Oncology, Charité University Hospital, Berlin, Germany
- Pediatric Hematology and Oncology, Hannover Medical School, Hannover, Germany

The consortium name UFO stands for the Umweltforschungsplan (UFOPLAN) of the Federal Ministry for the Environment, Nature Conservation, and Nuclear Safety (BMU), which granted funding through its Federal Office for Radiation Protection (FKZ 3612 S 70014). The I-BFM Study Group members are listed on the homepage
http://www.bfm-international.org/organization/members.php.

Supplementary Methods: Detailed Bioinformatic Workflow

1. Initial stringent alignment to hg19.

The paired-end reads are aligned to the host genome using `BWA` (`aln -n 2`), followed by `BWA's sampe` command. The SAM file must be retained for our Vy-PER pipeline, and also converted into sorted BAM format using SAMtools.

2. Extraction of partially unmapped read-pairs.

The unmapped end of a paired-end fragment which partly aligned to hg19 is extracted from the BAM file using `SAMtools` (`view -f 4 -F 264`). The resulting SAM file is converted to FASTA format using the Vy-PER script `vyper_sam2fas_se`.

3. Filtering of reads with less than 30 bp non-STR region.

`Phobos 3.3.12` is used for exact STR-typing of each read, and `vyper_sam2fas_se` is used to remove reads in which the main STR leaves less than 30 bp of remaining read. This remaining read may be an STR, but the read is nevertheless retained at this stage, as it is difficult to rule out that a unique alignment to a virus genome will not occur. The threshold of 30 bp can be changed by the user, but was chosen as the default, because 30 bp single-end reads align unambiguously to about 80% of the human genome [45]. The reads that are left after this filtering are converted to FASTA format using the Vy-PER script `vyper_sam2fas_se`.

4. Alignment to all NCBI virus genomes.

The extracted reads are mapped to the virus genome FASTA file with `BLAT (-out=blast8 -noTrimA -t=dna -q=dna -maxGap=0 -fastMap)` and the results are summarised in a table of virus candidates per genomic integration site using `Vy-PER_blatsam`. Only the top 3 virus candidates per integration site are retained. `Vy-PER_blatsam` also generates a folder of FASTA files that can be used with other alignment tools for optional manual inspection of each virus candidate. The tool `BLAT` is employed because `BWA` is not a sensitive aligner, even with the setting "`BWA -n 5`". In our tests, BLAT was in some cases more sensitive than `BLAST`, and when we tested their speed, the same 1000 sequences were aligned 7× faster by `BLAT`.

5. Final filtering of false positive virus candidates.

The Vy-PER script `Vy-PER_final_filtering` removes virus candidate sequences consisting mainly of an STR (default threshold: 50% of virus candidate sequence length), which stem from a more complex read that was not removed in step 3. The remaining sequences are aligned to the host genome reference sequence window expected for both paired-end reads (window size = 5 × read length), using `BLAT` with sensitive settings `(-out=blast8 -noTrimA -t=dna -q=dna -repMatch=1000000 -fine -tileSize=11 -stepSize=5)`. These settings make `BLAT` too slow for aligning low-complexity reads to the entire human genome. For instance, we aborted the `BLAT`-alignment of 770 such reads after 17.5 hours. For this reason `Vy-PER_final_filtering` outputs a FASTA file of virus candidates that can be filtered further using the FPGA-based Smith-Waterman aligner on the RIVYERA architecture. Furthermore, `Vy-PER_final_filtering` clusters virus candidates into genomic windows for graphical and tabular summary statistics. The default genomic window size is 1k bps, the minimal number of virus/host chimeras within the cluster is by default 10, and the minimal number of chimeras from the same virus within the cluster is by default 10. To increase sensitivity, the minimal number of chimeras can be reduced to 1. To reduce the number of genomic windows, their size can be increased without limit, e.g. to 1M bps.

6. Optional Smith-Waterman alignment to host genome.

The virus candidates in the FASTA file are aligned to hg19 with the FPGA-based Smith-Waterman aligner using the scoring matrix NUC44 (match: 5, mismatch: -4, gap open: -10, gap extension: -1) and requesting only one single best alignment. Vy-PER_final_filtering reads the Smith-Waterman aligner's out_detail.txt file and removes virus candidate sequences which aligned well to the host genome. Due to the strongly varying virus candidate sequence lengths, the alignment score does not allow a direct conclusion on whether a significant stretch of the read (default: 90% of the read length) was mapped (indel-free but permitting mismatches) to the host genome. Instead, we evaluated the CIGAR string of the alignment.

7. Results files.

The Vy-PER pipeline generates

a) an ideogram plot in PDF format giving a summary of candidate loci and virus types (see main manuscript, **Figure 1**),
b) a table of the top 10 virus candidates,
c) a table of the clusters (genomic windows, number of candidates, virus name and NCBI ID),
d) a table of phiX174 chimeras per chromosome (optical sequencing noise of the Illumina platform),
e) a detailed table of unfiltered virus candidates,
f) FASTA files for each virus candidate for optional manual alignment/checking.

Supplementary Results

True human sequence removed by DUST filtering in SURPI and BLAST

SURPI reported a file with unmapped reads for our test data set NA12878V. One of the first reads that was not mapped to the human genome or to any other genomes is:
@SRR010940.13891198/1
GACAGTTTGTTTGTTTGTTTGTTTGTTTGTTTGTTTTTTGAGATAGAGTCTTGCTCTGTTGCCC

BLAT correctly maps this read to human chromosome 22:

Human BLAT Results

BLAT Search Results

```
   ACTIONS      QUERY           SCORE START  END QSIZE IDENTITY CHRO STRAND  START    END       SPAN
---------------------------------------------------------------------------------------------------
browser details YourSeq           64     1    64    64 100.0%    22   -     16052140  16052203    64
browser details YourSeq           50     6    63    64  94.8%     1   +    161206753 161206817    65
```

Alignment of YourSeq and chr22:16052140-16052203

Click on links in the frame to the left to navigate through the alignment. Matching bases i
mark the boundaries of gaps in either sequence (often splice sites).

cDNA YourSeq

```
GACAGTTTGT TTGTTTGTTT GTTTGTTTGT TTGTTTTTTG AGATAGAGTC  50
TTGCTCTGTT GCCC
```

Genomic chr22 (reverse strand):

```
cttgggcaca tgttctcagg gtcttctgag ggctgtgtca tggaccatgg  16052254
tcattcatat ttggttcaga acaaatctct tcacatactg tacagatttt  16052204
GACAGTTTGT TTGTTTGTTT GTTTGTTTGT TTGTTTTTTG AGATAGAGTC  16052154
TTGCTCTGTT GCCCaggctg gagtgcaatg gcaaaatctc aggtcactgc  16052104
aacctccacc tcctgggttc aagcgattct cctgcctcag tctctgcagt  16052054
agctaagatt acag
```

Side by Side Alignment

```
00000001 gacagtttgtttgtttgtttgtttgtttgtttgttttttgagatagagtc 00000050
<<<<<<<< |||||||||||||||||||||||||||||||||||||||||||||||||| <<<<<<<<
16052203 gacagtttgtttgtttgtttgtttgtttgtttgttttttgagatagagtc 16052154

00000051 ttgctctgttgccc 00000064
<<<<<<<< |||||||||||||| <<<<<<<<
16052153 ttgctctgttgccc 16052140
```

BLAST incorrectly does not map this read at all:

BLAST ® Basic Local Alignment Search Tool
Home | Recent Results | Saved Strategies | Help
My NCBI [Sign In] [Regist

NCBI/ BLAST/ blastn suite/ Formatting Results - XR41DGK7015

Edit and Resubmit | Save Search Strategies | Formatting options | Download | YouTube How to read this page | Blast report description

Nucleotide Sequence (64 letters)

RID XR41DGK7015 (Expires on 08-02 21:03 pm)

Query ID lcl|42079
Description None
Molecule type nucleic acid
Query Length 64

Database Name Human G+T (2 databases)
Description See details
Program BLASTN 2.2.29+ Citation

No significant similarity found. For reasons why, click here

Other reports: Search Summary

Explanation: SURPI uses DUST to eliminate low complexity reads. BLAST also uses DUST and eliminated this real human read that was sequenced in the 1000 Genomes Project from the individual NA12878. Note that this read could be unambigously mapped to the human chromosome 22 by BWA.

Microhomologies: Three identical stretches between human and virus references in the 268T patient sequence at HBV integration locus chr19:30297359

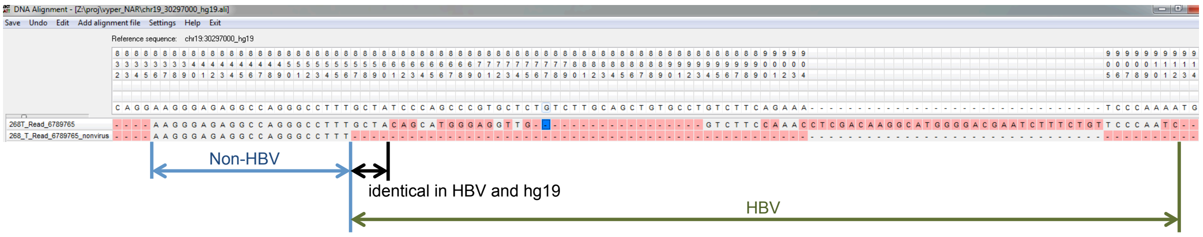

Using a visual inspection tool and Dialign-based alignment, we aligned the 101 bp read that BLAT mapped to HBV to the expected 1001 bp window of hg19 (upper row of sequences).

The aligned sequences are:

(a) the full human patient sequence that was partially mapped to HBV by BLAT (middle row with blue highlighted cell). The sequence stretch mapping to HBV ranges from column 857-910.

(b) the sequence stretch that was not mapped to HBV by BLAT (bottom row, columns 836-856). This stretch aligns perfectly to hg19, but the four next nucleotides (GCTA, columns 857-860) also map perfectly to hg19.

To clarify, a sequence stretch of four nucleotides is identical in the HBV part of the read, and in the hg19 reference.

It can be seen that two further stretches (columns 894-899, and columns 905-910) are identical between the HBV reference and the hg19 reference.

The four shared nucleotides in columns 857-860 appear to be consistent with random HBV integration into double strand breaks by the nonhomologous end joining (NHEJ) pathway, requiring microhomologies. The other end of the HBV integration shows a microhomology of five nucleotides (see page 8, **fusion read 2** at chr19:30298787). The computed HBV integration on chromosome 5 even shows a shared stretch of 13 nucleotides (see page 9, **Fusion reads 3 and 7 (identical)** at chr5:1292392), but no matching „other end“ for this HBV integration was detected.

We are not able to explain the other two identical stretches (columns 894-899 and 905-910), and they may just be a coincidence, although complex or repeated re-arrangements have been reported previously for HBV integrations.

N.B.: The coordinate numbering on chr19 is given in 1-based coordinates:
chromosomal position = column number + 30297000 – 500

Manual check of ViralFusionSeq HBV fusion reads for data set 268T:

Fusion read 1

GATTGGGAACAGAAAGATTCGTCCCCATGCCTTGTCGAGGTTTGGAAGACCAACCTCCCATGCTGTAGCAAAGGCCCTGGCCTCTCCCTTGATTGGGAACAGAAAGATTCGTCCCCATGCCTTGTCGAGGTTTGGAAGACCAACCTCCCATGCTGTAGCAAAGGCCCTGGCCTCTCCCTT

This is in fact an erroneous concatenation (by ViralFusionSeq) of two identical 90 bp reads.

The manually corrected fusion read is:
GATTGGGAACAGAAAGATTCGTCCCCATGCCTTGTCGAGGTTTGGAAGACCAACCTCCCATGCTGTAGCAAAGGCCCTGGCCTCTCCCTT

BLAT Search Results

ACTIONS	QUERY	SCORE	START	END	QSIZE	IDENTITY	CHRO	STRAND	START	END	SPAN
browser details	YourSeq	32	57	90	90	100.0%	3	-	168430628	168431213	586
browser details	YourSeq	25	66	90	90	100.0%	19	-	30297335	30297359	25

Alignment of YourSeq and chr19:30297335-30297359

Click on links in the frame to the left to navigate through the alignment. Matching bases in cDNA and genomic sequences are colored blue and capitalized. Light blue bases mark the boundaries of gaps in either sequence (often splice sites).

cDNA YourSeq

```
gattgggaac agaaagattc gtccccatgc cttgtcgagg tttggaagac  50
caacctccca tgctgTAGCA AAGGCCCTGG CCTCTCCCTT
```

Genomic chr19 (reverse strand):

```
tctgttctgt gtctgactgc cataccctca gctttcattt aaatctcatt  30297410
ttgggatttc tgaagacagg cacagctgca agacagagca cgggctggga  30297360
TAGCAAAGGC CCTGGCCTCT CCCTTcctgt cagggtccag cattttcctc  30297310
caagctacac cctctctgca ctttaatggg ggaggggcct tggccaatca  30297260
gaatcccgca ttcctggcca tgcga
```

Side by Side Alignment

```
00000066 tagcaaaggccctggcctctccctt 00000090
<<<<<<<< ||||||||||||||||||||||||| <<<<<<<<
30297359 tagcaaaggccctggcctctccctt 30297335
```

BLAT’s hg19 alignment is validated by Sung and colleagues [21] Suppl. Table S3: chr19:30297359 (Also validated by Vy-PER.The other read of the pair was confidently mapped to hg19 by BWA.)

BLASTN (v2.2.29) search results (top hit of 100 hits):

Hepatitis B virus isolate MY491357 polymerase (P) gene, complete cds
Sequence ID: gb|KJ717844.1| Length: 2532 Number of Matches: 1

Range 1: 534 to 602 GenBank Graphics ▼ Next Match ▲ Previous Match

Score	Expect	Identities	Gaps	Strand
125 bits(138)	5e-26	69/69(100%)	0/69(0%)	Plus/Minus

```
Query  1    GATTGGGAACAGAAAGATTCGTCCCCATGCCTTGTCGAGGTTTGGAAGACCAACCTCCCA  60
            ||||||||||||||||||||||||||||||||||||||||||||||||||||||||||||
Sbjct  602  GATTGGGAACAGAAAGATTCGTCCCCATGCCTTGTCGAGGTTTGGAAGACCAACCTCCCA  543

Query  61   TGCTGTAGC  69
            |||||||||
Sbjct  542  TGCTGTAGC  534
```

Fusion read 2

AAGTCAGAAGGCAAAAAAGAGAGTAACTCCACAGAAGGACAAATAACCCCCAAATCTCTTTATGAGTAATAACAACACAAAT
TGAGCAGTAAGTCAGAAGGCAAAAAAGAGAGTAACTCCACAGAAGGACAAATAACCCCCAAATCTCTTTATGAGTAATAAC
AACACAAATTGAGCAGT

The manually corrected fusion read is:
AAGTCAGAAGGCAAAAAAGAGAGTAACTCCACAGAAGGACAAATAACCCCCAAATCTCTTTATGAGTAATAACAACACAAAT
TGAGCAGT

BLAT Search Results

```
   ACTIONS         QUERY            SCORE START  END QSIZE IDENTITY CHRO STRAND  START      END       SPAN
---------------------------------------------------------------------------------------------------------
browser details YourSeq             51    40    90    90 100.0%    19   +   30298788 30298838     51
browser details YourSeq             31     8    55    90  97.0%    14   -   42428889 42428989    101
browser details YourSeq             21    42    63    90 100.0%     X   -   81085954 81085976     23
```

BLAT's hg19 alignment is validated by Sung *et al* Suppl. Table S3: chr19:30298787
(Not reported by Vy-PER, possibly because BWA was able to map a sub-stretch of each candidate read to hg19 and therefore there are no totally unmapped reads at this locus, only soft-clipped reads.)

BLASTN (v2.2.29) search results (top hit of 100 hits):

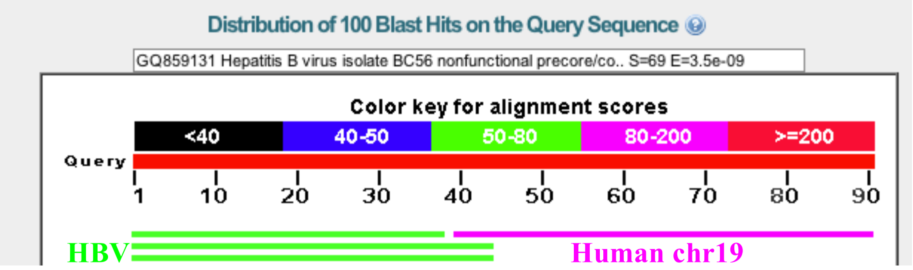

BLASTN shows a microhomology of five nucleotides (CAAAT) between hg19 and HBV:

Homo sapiens chromosome 19 clone CTD-2057D4, complete sequence
Sequence ID: gb|AC008798.10| Length: 135331 Number of Matches: 1

Range 1: 33897 to 33947 GenBank Graphics ▼ Next Match ▲ Prev

Score	Expect	Identities	Gaps	Strand
93.3 bits(102)	3e-16	51/51(100%)	0/51(0%)	Plus/Plus

```
Query  40     CAAATAACCCCCAAATCTCTTTATGAGTAATAACAACACAAATTGAGCAGT  90
              |||||||||||||||||||||||||||||||||||||||||||||||||||
Sbjct  33897  CAAATAACCCCCAAATCTCTTTATGAGTAATAACAACACAAATTGAGCAGT  33947
```

Download ⌄ GenBank Graphics

Hepatitis B virus isolate dxn1023, complete genome
Sequence ID: gb|KC774473.1| Length: 3215 Number of Matches: 1

Range 1: 1924 to 1968 GenBank Graphics ▼ Next Match ▲ Prev

Score	Expect	Identities	Gaps	Strand
69.8 bits(76)	3e-09	43/45(96%)	1/45(2%)	Plus/Minus

```
Query  1     AAGTCAGAAGGCAAAAAAGAGAGTAACTCCACAGAAG-GACAAAT  44
             ||||||||||||||||||||||||||||||||||||| | |||||
Sbjct  1968  AAGTCAGAAGGCAAAAAAGAGAGTAACTCCACAGAAGCGCCAAAT  1924
```

Fusion reads 3 and 7 (identical)

GCCTGGGAGCACTGGGAGCCAAAAGGTTAGTATCCCTTGGACTCATAAGGTGGGAAACTTTACTGGGCTTTATTCTTCTAC
TGTACCTGTGCCTGGGAGCACTGGGAGCCAAAAGGTTAGTATCCCTTGGACTCATAAGGTGGGAAACTTTACTGGGCTTTA
TTCTTCTACTGTACCTGT

The manually corrected fusion read is:
GCCTGGGAGCACTGGGAGCCAAAAGGTTAGTATCCCTTGGACTCATAAGGTGGGAAACTTTACTGGGCTTTATTCTTCTAC
TGTACCTGT

BLAT Search Results

```
   ACTIONS      QUERY           SCORE START  END QSIZE IDENTITY CHRO STRAND  START     END      SPAN
---------------------------------------------------------------------------------------------------
browser details YourSeq            26     1    26    90 100.0%     5   +    1292367   1292392     26
```

cDNA YourSeq

```
GCCTGGGAGC ACTGGGAGCC AAAAGGttag tatcccttgg actcataagg  50
tgggaaactt tactgggctt tattcttcta ctgtacctgt
```

Genomic chr5 :

```
gaaggtcacc ctccttgtct gcatggccgg aagtcttaca tgtcttggga  1292316
gtttgtgggg agggggtgaa atcgggactt cttctagctg ccacggtagg  1292366
GCCTGGGAGC ACTGGGAGCC AAAAGGgggc tggagcggag gttcctcaac  1292416
atcaaatcca gaaaaacagg gtggggacac ggcagggccc agcagcacca  1292466
tcccctgaac acccacaaac actgtc
```

Side by Side Alignment

```
0000001 gcctgggagcactgggagccaaaagg 0000026
>>>>>>> |||||||||||||||||||||||||| >>>>>>>
1292367 gcctgggagcactgggagccaaaagg 1292392
```

BLAT's hg19 alignment is validated by Sung and colleagues [21] Suppl. Table S3: chr5:1292392 (not reported by Vy-PER)

BLASTN (v2.2.29) search results (top hit of 100 hits) show a **near-identical stretch of 13 nucleotides** (GGGAGC ACTGGGAGCC AAAAGG) between hg19 and HBV:

Hepatitis B virus isolate MY491357 polymerase (P) gene, complete cds
Sequence ID: gb|KJ717844.1| Length: 2532 Number of Matches: 1

Range 1: 130 to 206 GenBank Graphics ▼ Next Match ▲ Previ

Score	Expect	Identities	Gaps	Strand
122 bits(134)	6e-25	73/77(95%)	0/77(0%)	Plus/Plus

```
Query  14   GGGAGCCAAAAGGTTAGTATCCCTTGGACTCATAAGGTGGGAAACTTTACTGGGCTTTAT  73
            |||| ||  || ||||||||||||||||||||||||||||||||||||||||||||||||
Sbjct  130  GGGAACCTCAATGTTAGTATCCCTTGGACTCATAAGGTGGGAAACTTTACTGGGCTTTAT  189

Query  74   TCTTCTACTGTACCTGT  90
            |||||||||||||||||
Sbjct  190  TCTTCTACTGTACCTGT  206
```

Fusion read 4

CCTGACAGGAAGGGAGAGGCCAGGGCCTTTGCTACAGCATGGGAGGTTGGTCTTCCAAACCTCGACAAGGCATGGGGAC
GAATCTTTCTGCCTGACAGGAAGGGAGAGGCCAGGGCCTTTGCTACAGCATGGGAGGTTGGTCTTCCAAACCTCGACAAG
GCATGGGGACGAATCTTTCTG

The manually corrected fusion read is:
CCTGACAGGAAGGGAGAGGCCAGGGCCTTTGCTACAGCATGGGAGGTTGGTCTTCCAAACCTCGACAAGGCATGGGGAC
GAATCTTTCTG

BLAT Search Results

ACTIONS	QUERY	SCORE	START	END	QSIZE	IDENTITY	CHRO	STRAND	START	END	SPAN
browser details	YourSeq	36	3	52	90	95.2%	15	-	100001092	100001193	102
browser details	YourSeq	34	1	34	90	100.0%	19	+	30297326	30297359	34
browser details	YourSeq	27	16	43	90	100.0%	3	+	168430819	168431213	395
browser details	YourSeq	20	7	26	90	100.0%	20	+	9294686	9294705	20

BLAT's hg19 alignment is validated by Sung and colleagues [21] Suppl. Table S3: chr19:30297359 (Also validated by Vy-PER.The other read of the pair is confidently mapped to hg19 by BWA.)

BLASTN (v2.2.29) search results (top hit of 100 hits):

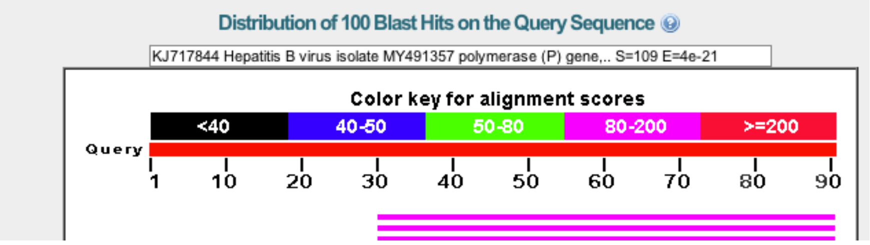

Fusion reads 5 and 6 (identical)

CAGAAAGATTCGTCCCCATGCCTTGTCGAGGTTTGGAAGACCAACCTCCCATGCTGTAGCAAAGGCCCTGGCCTCTCCCT
TCCTGTCAGGCAGAAAGATTCGTCCCCATGCCTTGTCGAGGTTTGGAAGACCAACCTCCCATGCTGTAGCAAAGGCCCTG
GCCTCTCCCTTCCTGTCAGG

The manually corrected fusion read is:
CAGAAAGATTCGTCCCCATGCCTTGTCGAGGTTTGGAAGACCAACCTCCCATGCTGTAGCAAAGGCCCTGGCCTCTCCCT
TCCTGTCAGG

BLAT Search Results

ACTIONS	QUERY	SCORE	START	END	QSIZE	IDENTITY	CHRO	STRAND	START	END	SPAN
browser details	YourSeq	36	39	88	90	95.2%	15	+	100001092	100001193	102
browser details	YourSeq	34	57	90	90	100.0%	19	-	30297326	30297359	34
browser details	YourSeq	27	48	75	90	100.0%	3	-	168430819	168431213	395
browser details	YourSeq	20	65	84	90	100.0%	20	-	9294686	9294705	20

BLAT's hg19 alignment is validated by Sung and colleagues [21] Suppl. Table S3: chr19:30297359 (Also validated by Vy-PER.The other read of the pair is confidently mapped to hg19 by BWA.)

BLASTN (v2.2.29) search results (top hit of 100 hits):

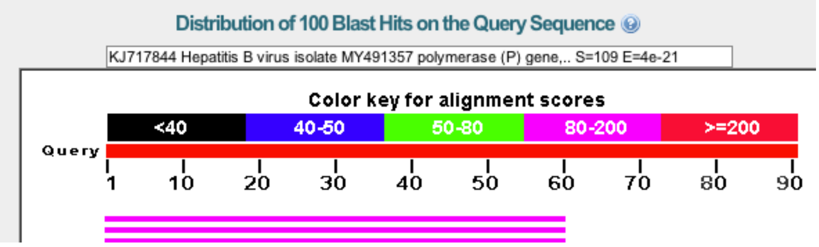

Supplement: Supplementary Information [file srep11534-s1.pdf]
